# Influence of Catalyst Content and Epoxy/Carboxylate Ratio on Isothermal Creep of Epoxy Vitrimers

**DOI:** 10.3390/polym15183845

**Published:** 2023-09-21

**Authors:** Barbara Palmieri, Fabrizia Cilento, Eugenio Amendola, Teodoro Valente, Stefania Dello Iacono, Michele Giordano, Alfonso Martone

**Affiliations:** 1 Institute of Polymers, Composite and Biomaterials (IPCB), National Research Council of Italy, 80055 Portici, Italy; barbara.palmieri@ipcb.cnr.it (B.P.); fabrizia.cilento@ipcb.cnr.it (F.C.); stefania.delloiacono@cnr.it (S.D.I.); michele.giordano@cnr.it (M.G.); alfonso.martone@cnr.it (A.M.); 2 Agenzia Spaziale Italiana (ASI), Via del Politecnico snc, 00133 Roma, Italy; teodoro.valente@uniroma1.it

**Keywords:** compression molding, vitrimer, multifunctional composites, epoxy matrix

## Abstract

In the present work, a commercial epoxy based on epoxy anhydride and tertiary amine was modified by a metallic catalyst (Zn^2+^) to induce vitrimeric behavior by promoting the transesterification reaction. The effect of two different epoxy/acid ratios (1 and 0.6) at two different zinc acetate amounts (Zn(Ac)_2_) on the thermomechanical and viscoelastic performances of the epoxy vitrimers were investigated. Creep experiments showed an increase in molecular mobility above the critical “Vitrimeric” temperature (T_v_) of 170 °C proportionally to the amount of Zn(Ac)_2_. A procedure based on Burger’s model was set up to investigate the effect of catalyst content on the vitrimer ability to flow as the effect of the dynamic exchange reaction. The analysis showed that in the case of a balanced epoxy/acid formulation, the amount of catalyst needed for promoting molecular mobility is 5%. This system showed a value of elastic modulus and dynamic viscosity at 170 °C of 9.50 MPa and 2.23 GPas, respectively. The material was easily thermoformed in compression molding, paving the way for the recyclability and weldability of the thermoset system.

## 1. Introduction

Thermosets are widely used in the composite industry. Despite their structural performances, their recyclability remains a global challenge. Thermoset polymers are characterized by a stable cross-linked structure between polymer chains that enables high-temperature resistance, high thermal and dimensional stability, good resistance to solvent, and excellent mechanical properties [1,2,3].

Nevertheless, the strong polymer network hinders the essential long-range molecular mobility needed to initiate material flow at elevated temperatures for reformation and recycling [4,5]. As a result, recycling thermoset polymers and composites is a challenging task, and the majority of their components are disposed of in landfills when their useful lifespan concludes, leading to a significant adverse effect on the environment [6]. The growing environmental concerns and heightened industrial competition are driving the promotion and advancement of structural materials that can be repaired and recycled. This movement aims to mitigate the generation of polymer waste and extend the service life of these materials [7].

Nowadays, several methods have been developed to recycle thermoset composites based on mechanical, chemical, and thermal processes [8]. The mechanical recycling process consists of shredding procedures aimed at reducing the size of composite components to make them recyclable. The thermal recycling process of carbon fiber composite material consists of burning the material in the absence/existence of oxygen to degrade the matrix and extract the fibers [9]. Finally, the chemical recycling process consists of using different supercritical fluids and catalytic solutions to separate the oligomers obtained by the decomposition of polymeric resin that can be reused as chemical raw materials and carbon fibers.

Nevertheless, none of these processes meet sustainability requirements [10]. According to the EU guidelines for waste management [11], waste prevention represents the most efficient way to recycle material.

A strategy to preserve the advantages of thermosetting polymers able to enable recyclability, weldability, and reparability is to modify the network by cleavable or dynamics bonds [12]. The development of Covalent Adaptable Networks (CANs) is a viable step towards producing recyclable epoxy resins to be employed in composite components that can be recycled and reformed as thermoplastic materials [13,14]. CANs are characterized by covalent crosslinks that become reversibly dynamic under specific external stimuli (i.e., heat, catalyst, light) [15,16].

CANs can be divided into dissociative and associative covalent adaptive networks based on the mechanism of reversible chemical bond exchange [17]. The first one can be considered depolymerization, consisting of a topological reshuffling of polymeric networks that allows to rework and reform of the cross-linked material [15,18]. Under an external stimulus, the entire dissociative CAN (dCAN) networks break faster than they reform, resulting in an increase in the polymer mobility due to the reduction of the crosslinking density. When the external stimulus is removed, the crosslinking density of dCAN increases, recovering the mechanical properties, eventually reaching the same level as that of the initial state [19]. One of the most studied dCAN reaction mechanisms is the Diels-Alder [16,20,21,22].

**Figure 1:**
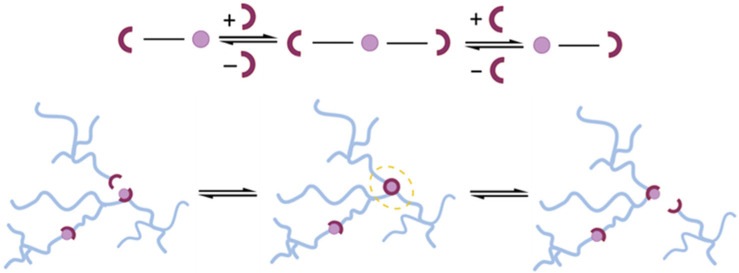
Exchange reaction scheme and related topological changes. In the associative bond-exchange reaction, the overall crosslink density remains constant; the crosslinks are broken only when new ones are created (highlighted by the yellow dotted line) without resulting in a loss of crosslinks at the previous position.

In contrast, the associative CANs (aCAN) are characterized by constant crosslinking density during the exchange reaction, breaking old bonds and generating new bonds simultaneously (Figure 1). The pioneers of aCANs were Leibler and co-workers in 2011 [23], who applied the well-established transesterification reaction to the hydroxyl and ester groups in an anhydride-cured epoxy matrix to create a reworkable thermosetting network named vitrimers. During transesterification, the network’s connectivity is altered via exchange reactions, inducing stress relaxation and plastic flow at elevated temperatures without depolymerization [24].

Vitrimers behave differently according to the service temperature: below the topology freezing transition temperature (T_v_), vitrimers behave like traditional thermosets, showing good thermal and mechanical properties; above the T_v,_ they flow like viscoelastic fluid thanks to the topology rearrangement induced by transesterification reactions. [13,23].

Epoxy vitrimers based on transesterification reactions are the most studied; the exchange reactions between esters and beta-hydroxyls formed by reacting epoxy precursors with suitable acids/anhydrides are exploited [13,25]. The acceleration of the transesterification reactions is governed by the incorporation of a catalyst. The topological variations, stress relaxation, and flow are induced in the cross-linked networks, without changing the total number of crosslinks [26,27].

Various catalysts, including metal-containing compounds and organocatalysts, are explored to promote transesterification and curing in epoxy vitrimer production [28,29,30]. Zinc ions (Zn^2+^) modify epoxy reactivity, opening anhydride rings and yielding monoesters and carboxylic acids. This acid then reacts with epoxy, forming diesters and β-hydroxyl ester chains [13,25]. Catalyst Zn(Ac)_2_ significantly accelerates esterification during curing. It is noted that Zn(Ac)_2_ drives the dynamic transesterification in epoxy-based vitrimer resin [13,23,27,31]. Yang et al. [32] incorporated transesterification into the anhydride/epoxy system, yielding repairable hard epoxy with 2% mol of zinc acetylacetonate (Zn(acac)_2_). Shi et al. [33] studied weldability and reprocessing, and Demongeot et al. [34] explored the mechanism of catalytic zinc species in these materials.

When the reaction temperature is above the topological freezing transition temperature (T_v_), free hydroxyl groups induce dynamic transesterification reactions in the cross-linked ester groups under the action of catalyst Zn (Ac)_2_. This leads to changes in the topology of the cross-linked network while maintaining a constant density of the network and allowing rearrangements that result in a macroscale flow [35,36].

The network structure of epoxy vitrimers is directly affected by the chemical structure of the curing agent and the epoxide equivalent of epoxy oligomers. In order to achieve a good compromise between the thermo-mechanical properties and a sufficient amount of free hydroxyl groups available for transesterification, a tuning of the epoxy-to-carboxyl ratio is required [37,38,39,40].

For this study, a commercially available epoxy system (ARALDITE^®^ LY 3508 and ARADUR^®^ 917-1 developed by Huntsman Corporation, The Woodlands, TX, USA), commonly used in the manufacturing of Carbon Fiber Reinforced Polymers (CFRPs), was chosen to transform it into a vitrimeric system. Two formulations with different stoichiometric ratios between the epoxy precursor and the curing agent were formulated and subsequently subjected to modifications by adding Zinc acetate Zn(Ac)_2_ as a transesterification catalyst. The effect of the amount of Zn(Ac)_2_ on the thermal and thermo-mechanical properties of epoxy vitrimeric systems was investigated. Creep experiments were performed to assess the vitrimeric behavior, showing a flow above a critical temperature (about 170 °C). Finally, the recyclability of the vitrimeric system and its thermoformability were also assessed.

## 2. Materials and Methods

### 2.1. Materials and Epoxy Vitrimer Formulation

The resin utilized in this study comprises a mixture of Bisphenol A diglycidyl ether (DGEBA) epoxy resin, characterized by an Epoxy Equivalent Weight (EEW) of 196.5 g/eq. The curing agent employed was tetrahydro-methyl phthalic anhydride (THMPA), and the catalyst utilized was 2,4,6-tris(dimethylaminomethyl)phenol, provided under the product names ARALDITE^®^ LY 3508, ARADUR^®^ 917-1, and Accelerator 960-1, respectively, by Huntsman Corporation. Additionally, anhydrous zinc acetate Zn(Ac)_2_ (99.99% purity) was used as the catalyst, procured from Merck Sigma-Aldrich. All chemicals were utilized without undergoing further purification procedures.

Two different systems were prepared: (A) an epoxy mixture system, with a stoichiometric ratio of epoxy/acyl equal to 1, and (B) an epoxy mixture system, with a stoichiometric ratio of epoxy/acyls equal to 0.6. Two different vitrimeric formulations for each system were realized, varying ZnAc_2_ percentage between 5% and 10% with respect to the total acyl groups, as listed in Table 1.

**Figure 2:**
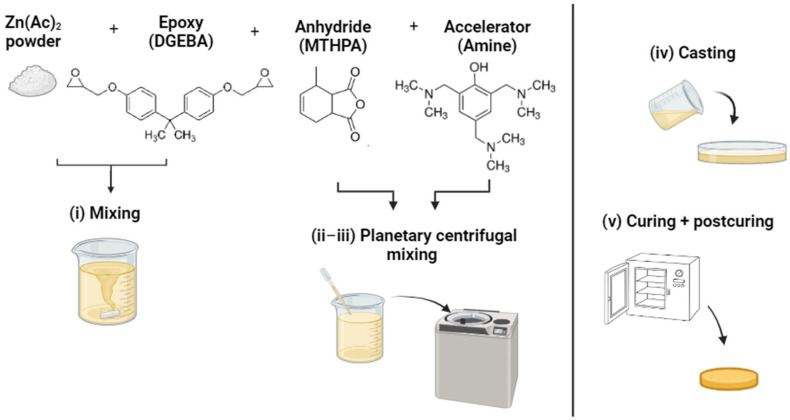
Fabrication procedure of vitrimeric epoxy resin.

The formulations were prepared following the same procedure, as illustrated in Figure 2: (i) hand-mixing of epoxy resin ARALDITE^®^ LY 3508 and zinc acetate fine powder (20 µm); (ii) addition of cross-linking anhydride ARADUR^®^ 917-1 and mixing with the planetary centrifugal mixer (THINKY mixer ARV 310) under vacuum at room temperature; (iii) addition of the Accelerator 960-1 and further mixing as in the previous step to obtain a homogeneous mixture; and (iv) casting in Teflon molds.

The addition of the tertiary amine to the epoxy ring with the formation of a Zwitterion is the first step of the crosslinking reaction of DGEBA with MTHPA. In a later stage, the alkoxide reacts with the carboxylic anhydride with the formation of a new –COO- group. The alternating addition of DGEBA and MTHPA builds up the network.

The ZnAC2 does not enter the network structure, even if the resulting Zn^2+^ ion can act as a further booster of the addition reaction between epoxy and anhydride. After crosslinking, the Zn^2+^ ions act as a catalyst for the ester interchange reaction, which is responsible for network mobility that allows the vitrimeric behavior but does not contribute to the network structure [41].

Finally, samples were cured for 1 h 30 min at 120 °C and post-cured for 2 h at 140 °C in the oven.

### 2.2. Experimental Characterization

Thermogravimetric analysis (TGA) was performed using a TA Instruments Q500 instrument (New Castle, DE, USA) to assess the thermal stability range of the polymer according to the ASTM E1131 standard [42]. The measurements were conducted under an inert atmosphere of nitrogen gas, with a temperature ramp of 10 °C/min from room temperature to 800 °C. The weight loss was quantified at 800 °C.

The thermal characteristics of the polymer were examined through differential scanning calorimetry (DSC) utilizing a TA Instruments Discovery DSC instrument. Each test specimen underwent two heating and cooling cycles from 0 to 250 °C at a rate of 10 °C/min in a nitrogen atmosphere. Before measurements, approximately 10 mg of the samples were enclosed in aluminum pans.

The glass transition temperature (Tg) and the enthalpy of the reaction were derived from the DSC curves in accordance with ASTM D3418 standards [43].

Dynamic mechanical analysis (DMA) was performed with a Dynamic Mechanical Analyzer Q800 from TA Instruments in the Single Cantilever mode (SC). Samples of a rectangular shape 25 mm in length, 5.5 mm in width, and about 2.5 mm in thickness were tested. The behavior of the polymer with temperatures between 30 and 180 °C was investigated considering a heating rate of 3 °C/min, a strain amplitude of 15 μm, and a frequency of 1 Hz. Data were elaborated according to the ASTM D790 standard [44] for the flexural behavior of unreinforced and reinforced plastics.

Tensile tests were performed with an Instron 68TM-50 universal testing apparatus. Dogbone samples of 2.5 mm thickness were tested at room temperature with a displacement rate of 2 mm/min. Data were elaborated according to the ASTM D638 standard [45] for plastic tensile strength tests.

Creep tests were performed using the DMA Q800 from TA Instruments (New Castle, DE, USA) equipped with the Tension Film (TF) clamp. Rectangular specimens of 10 mm × 5.5 mm × 2 mm were tested. Tests were performed at different temperatures from 70 °C to 245 °C, with 25 °C incremental steps to evaluate the sample strain variation from the glassy to the rubbery state. For each step, samples were isothermally held for five minutes, and then a constant stress of 0.1 MPa was applied for 45 min.

## 3. Thermomechanical Characterization of Epoxy Vitrimers

### 3.1. Thermal Characterization

**Figure 3:**
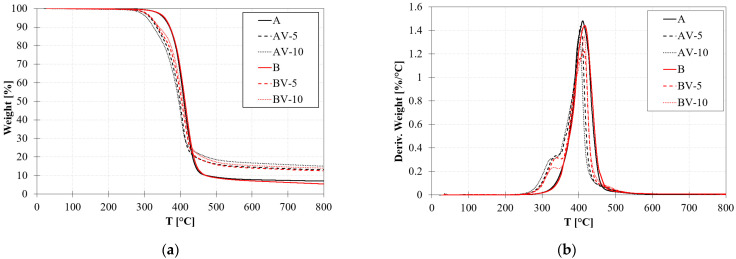
TGA thermograms: (**a**) weight loss and (**b**) weight derivatives.

Figure 3a,b shows the TGA thermograms of selected samples and their derivatives. Non-vitrimeric systems, A and B, almost have similar degradation profiles. The main decomposition of all samples occurs at around 410 °C (Table 2). In the case of vitrimeric polymers, a small weight loss of 10% (Table 2) was also found, associated with zinc acetate degradation. By increasing the amount of zinc acetate, a small reduction in the maximum decomposition temperature occurs. This phenomenon is likely related to the lower temperature of degradation of zinc acetate (269 °C, [46]), which is lower than that of the main degradation of the polymer; therefore, the mixture experiences degradation at lower temperatures. The char yield at 800 °C is reported in Table 2. It increases with increasing zinc acetate percentage, according to the amount of zinc acetate powder.

**Figure 4:**
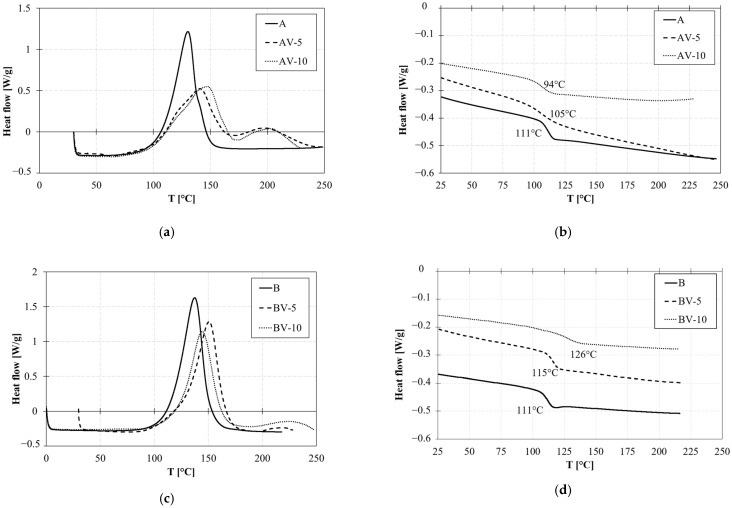
DSC curves of the sample (**a**) I scan of system A; (**b**) II scan of system A; (**c**) I scan of system B; and (**d**) II scan of system B.

The curing behavior and glass transition temperature of the systems are illustrated in Figure 4. The cure of all the tested systems is illustrated in Figure 4a for system A and Figure 4c for system B (first scan of the DSC Thermogram). In the case of non-vitrimetic systems, only one exothermic peak was found at 130 °C and 137 °C, respectively, for A and B. Whereas, in the case of vitrimeric systems, two exothermic peaks are observed: the first at around 140 °C and the second at 180 °C for AV-5, -10 and 230 °C for BV-5,-10. The first peak is related to the ester chain formation (esterification reaction) during cure, whereas the second peak is related to the homomeric ring-opening polymerization of the leftover epoxy groups. The B-System contains additional anhydrides able to react, leading to a single reaction peak. The presence of zinc acetate therefore influences the cross-linking reaction; in fact, a decrease in total enthalpy is observed with respect to the bare system, and a secondary reaction occurs in the range between 180 °C and 230 °C. Similar effects were observed for both systems, even if in the case of B formulation, the secondary reaction was negligible.

In the second heating ramp (Figure 4b–d), the absence of an exothermic peak indicates a complete polymer conversion. The glass transition temperature (T_g_) of both systems A and B is 111 °C. It drops in the case of the vitrimeric formulations AV-5 and -10 due to a lower cross-linking density. On the contrary, it increases in the case of the vitrimeric formulations BV-5 and -10.

Table 2 summarizes the thermal behavior of the analyzed systems characterized by TGA and DSC experiments.

### 3.2. Static and Dynamic Mechanical Characterization

**Figure 5:**
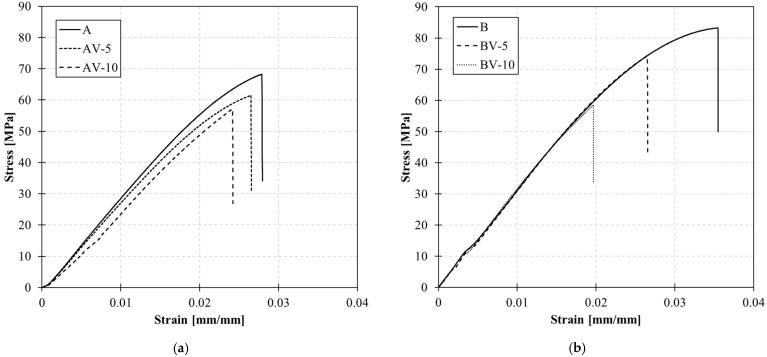
Tensile test on system A (**a**) and system B (**b**).

Figure 5 reports the stress-strain curve of the tensile test conducted on the samples. The ultimate stress and strain and the elastic modulus computed in the elastic range (between 0 and 0.005 mm/mm) are listed in Table 3. The chemically balanced (stoichiometric) formulation leads to a decrease in the ultimate strength; the higher the zinc acetate content, the lower the strength. In addition, the zinc acetate also affects the polymer’s elastic performance. The entanglement of zinc within the molecular topology affects the system stiffness, and Young’s modulus decreases according to the catalyst content. The effect of the catalyst content on the system mobility is showed also in the glass transition temperature; indeed, the T_g_ decreases according to the zinc concentration.

Conversely, the system made by unbalancing the reactant content achieves stiffness stability with Young’s modulus remaining almost constant. In the present case (B-formulation), the catalyst acts as a stiff inclusion within the network, contributing to reducing the system mobility. In this case, the glass transition increases according to the catalyst content. In both cases, the presence of the catalyst threatens the ultimate strength.

**Figure 6:**
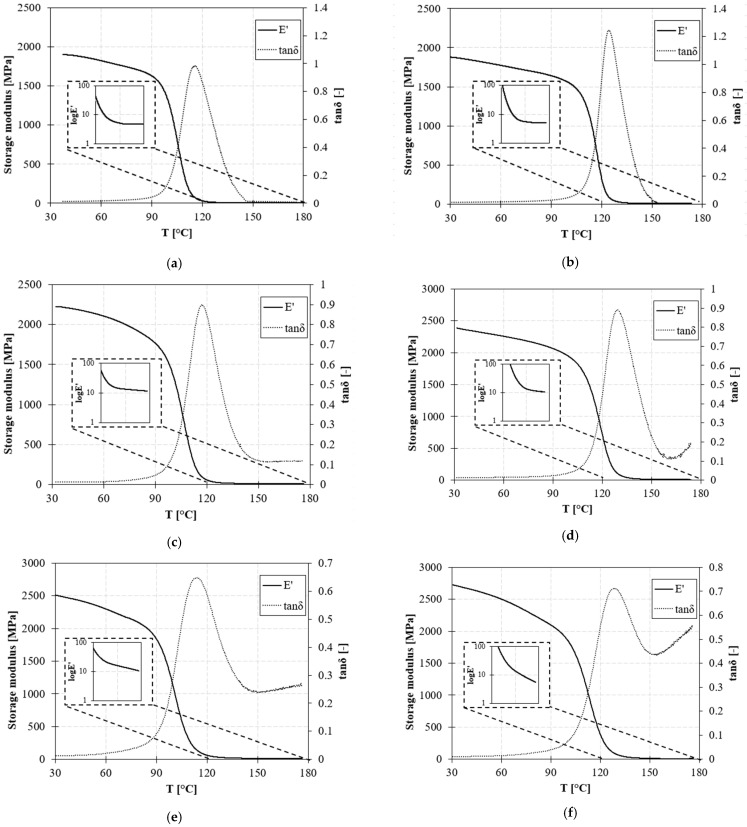
DMA curves of the sample (**a**) A; (**b**) B; (**c**) AV-5; (**d**) BV-5; (**e**) AV-10; and (**f**) BV-10. The first row shows the conventional epoxy system, and the subsequent rows describe the mobility induced by the catalyst effect at different content levels.

The viscoelastic behavior of the polymers with temperatures up to 180 °C is reported in Figure 6. DMA experiments were performed in the linear viscoelastic deformation range to confirm cross-linked samples’ glass transition temperatures and elastic modulus.

The glass transition temperature increases by 10 °C from system A to system B. However, no significant variations are observed when zinc acetate is added to the formulation.

The storage modulus at room temperature of non-trimeric epoxy systems A and B are the same. The addition of the Zn^2+^ catalyst produces a slight increase in the elastic modulus in both cases. In system A, an increase of 17% and 31% was obtained for vitrimeric formulations with 5% and 10% of zinc acetate, respectively. A higher increase is found in system B: 27% for sample BV-5 and 45% for sample BV-10.

The dissipation capacity, in terms of loss modulus (E″), also increases with the addition of zinc acetate. The enhancement is more pronounced in the case of system A, where E″ increased by +29% in AV-5 and +51% in AV-10. A lower increase of +18% is found in BV-5 and +15% in BV-10. Consequently, improvements in the loss factor are found only in system A, with a maximum of +16% for the sample with 10% of ZnAc_2_.

At a higher temperature of 170 °C, the loss moduli of the vitrimeric systems significantly increase compared to the non-vitrimeric systems, showing the high mobility of the polymer induced by the catalyst (Table 4). This effect becomes more pronounced with the increase in zinc acetate percentage.

### 3.3. Isothermal Creep of Zn^2+^ Epoxy Vitrimers

Creep experiments are an explicit and robust confirmation of the vitrimeric behavior of the resin due to thermoreversible cross-linking rearrangements when a constant load is applied.

As expected, the sample with the non-vitrimeric formulation (A and B) showed stable behavior; in fact, a negligible increase in strain was observed at increasing temperatures, showing the typical thermosetting behavior. Even at temperatures higher than T_g_, no molecular flow was observed.

**Figure 7:**
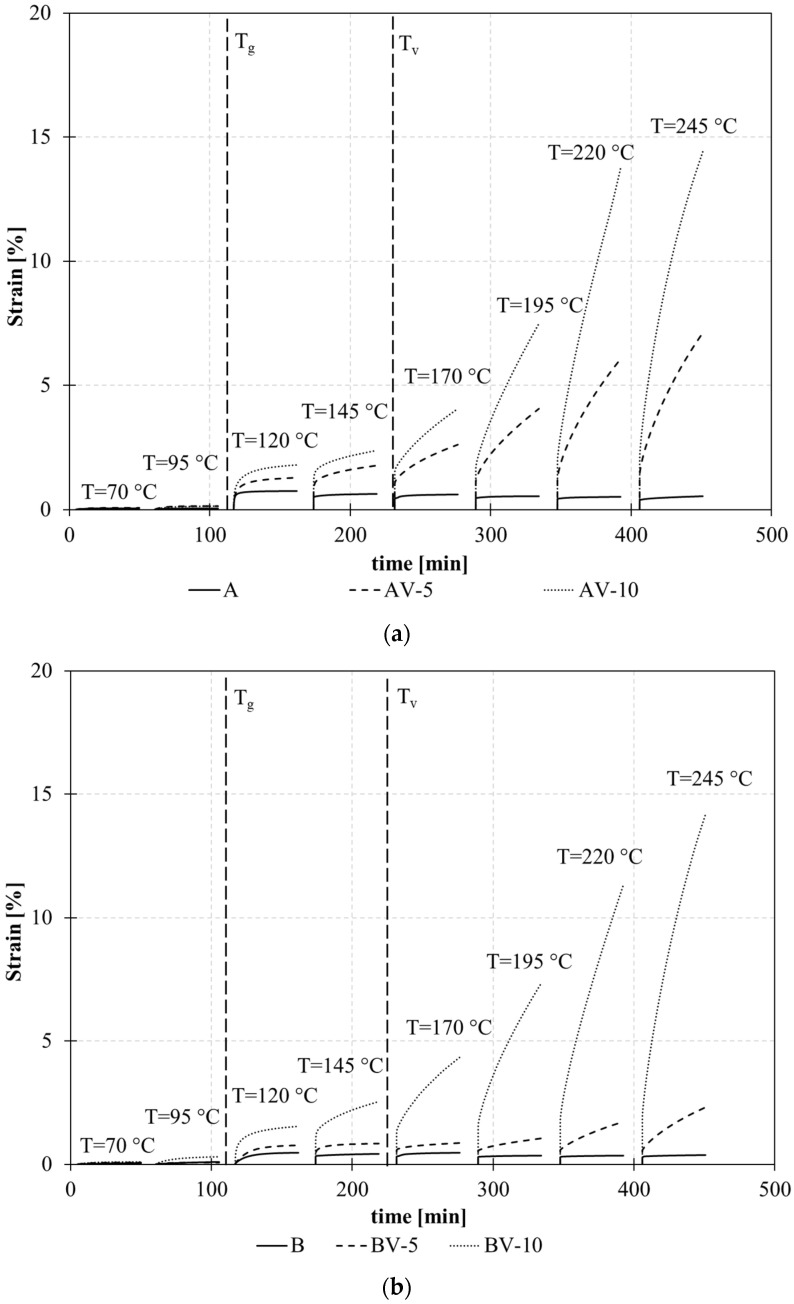
Creep curves at different temperatures of systems A (**a**) and B (**b**).

On the contrary, the vitrimeric formulations, both in the case of systems A and B, show a significant increase in strain under constant load at high temperatures above T_g_. The presence of metallic ions strongly catalyzes the ester interchange reaction, resulting in an evident creep of samples subjected to tensile load at different temperatures, as reported in Figure 7a,b. The effect of the amount of zinc acetate is also shown, since at temperatures greater than 170 °C, a significant molecular flow is observed. This value represents the topological freezing transition temperature (T_v_). In particular, the higher the zinc acetate percentage, the higher the measured strain under constant load.

## 4. Assessment of the Recyclability and Reshaping (3R Analysis)

The study of Recyclable, Repairable, and Reshapable (3R) for epoxy vitrimer allows a comprehensive evaluation of the material’s sustainability and potential for circular economy practices. The recyclability aspect of epoxy vitrimer, namely the efficiency and effectiveness of its recycling processes, include the ease of separating the material from other components, the energy requirements for recycling, and the quality of the recycled material obtained.

Furthermore, the repairability aspect focuses on the material’s ability to be restored after damage. Lastly, the reshaping aspect investigates the material’s capability to be molded or reshaped into different forms or products. This includes evaluating the ease of reshaping, the quality of the reshaped material, and the potential limitations or challenges in the process [47].

### 4.1. Mechanical Recycling

The ability to undergo a dynamic transesterification reaction gives the epoxy-based vitrimer resin the ability to be reshaped and reprocessed. The repairability and reuse capability of vitrimers were assessed in the case of AV-5 formulation, based on a trade-off between mechanical properties and dynamic properties. Among the analyzed formulations, the AV5 system exhibited a higher ability to flow, as demonstrated by creep analysis. In addition, with respect to the AV10, there was a slight change in the glass transition temperature with respect to the non-catalyzed system (DMA analysis).

**Figure 8:**
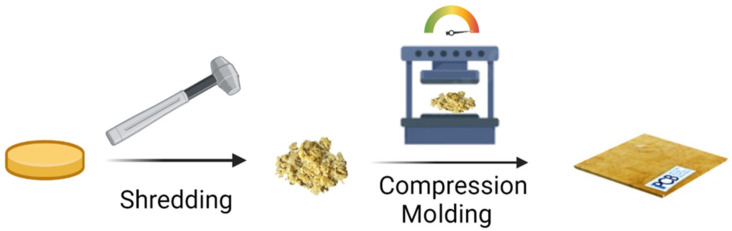
Recycling procedure of the vitrimeric epoxy AV-5.

The cured resin was shredded to a coarse powder (average size 500 microns), which was then compacted in a platen hot press by applying 10 bar at 200 °C for 30 min. The schematic of the recycling sequence is described in Figure 8. At the end of the molding process, a thin plate of 0.250 mm is obtained.

The use of zinc acetate as a catalyst enables the recyclability of the material, thanks to the vitrimeric nature of the system. The resin granules were able to fuse and be remolded due to the movement of the polymer chain between the interfaces under a dynamic transesterification reaction.

**Figure 9:**
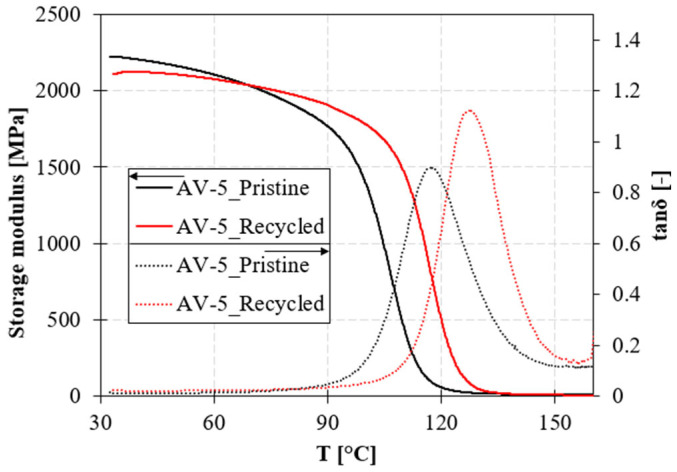
Results of DMA on recycled and pristine AV-5.

The mechanical properties of the recycled resin were assessed by DMA analysis and compared to the pristine material. As shown in Figure 9, the recycling procedure does not significantly affect the mechanical stiffness compared to the pristine resin; the storage modulus reduces by 5%. The tanδ increases due to the increase in the dissipative area at the contact between adjacent fused granules; in addition, the possible presence of non-perfect cohesion at the granules’ interfaces will increase the dissipative behavior. On the contrary, the T_g_ increases by 8 °C; the rationale for the increase is that the unreacted groups (irreversible chains) are cured during the second processing stage.

### 4.2. Composites Thermoforming

**Figure 10:**
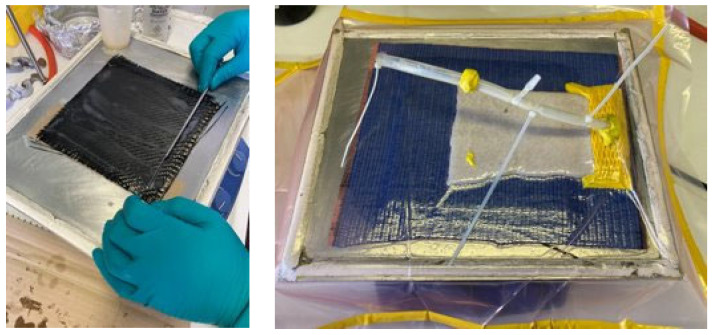
The manufacturing procedure for vitrimer-CFRP.

In order to verify the capability of the vitrimer to thermoform, a carbon fiber-reinforced composite plate was manufactured. Vitrimeric epoxy resin (AV-5) was employed to impregnate carbon fiber fabric 3k T300 Twill 2 × 2 (Toray). The laminate was manufactured by hand lay-up and vacuum consolidation at 1 h 30 min at 120 °C and post-cured 2 h at 140 °C in the oven (Figure 10).

**Figure 11:**
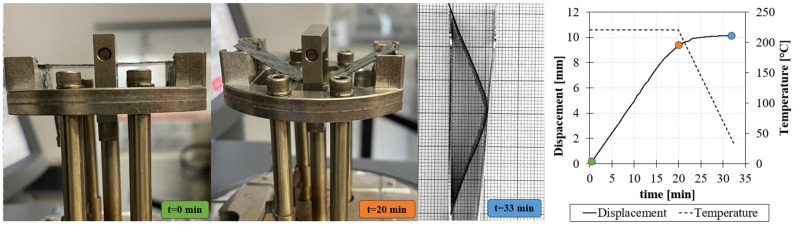
Experimental set-up and three-point bending thermoforming test procedure.

A CFRP layer (470 µm thick) was cut to fit the three-point bending fixture of a DMA instrument (dimensions 60 × 15 mm). The thermoforming was simulated by warming the sample within the DMA chamber up to 220 °C. The forming temperature was kept constant for 1 min to achieve homogenous heating of the specimens. Then, the specimens were deflected with a displacement speed of 500 μm/min up to 10 mm of maximum deflection point (Figure 11). After deflection, the specimens were cooled to room temperature at a rate of 15 °C/min and then removed from the clamp. After cooling, the sample maintained the imposed deflection without a spring-back effect.

Figure 11 shows the displacement induced on the sample, the temperature during the test, and the initial and final configurations. The test successfully confirms the thermoformability and that the reinforcement does not inhibit the resin flow and the subsequent reshaping capability.

## 5. Discussion

### 5.1. Effect of Catalyst Content

Creating cross-linked systems featuring semi-flexible molecular structures enables the occurrence of topological interchange reactions. Adjusting the catalyst content in the formulation allows for the management of the reactivity of interchange linkages and the thermomechanical characteristics.

The cross-linking of bifunctional epoxy precursors with monocarboxylic anhydrides is a widely recognized process that can be facilitated using either an excess or a deficiency of the curing agent [39]. Typically, this reaction is catalyzed by the inclusion of substituted amines. When a stoichiometric balance between epoxy and acyl groups is maintained, the resulting cross-linked structure possesses a lower density. However, the presence of hydroxyl and ester groups significantly promotes the occurrence of ester-interchange reactions [32]. The transesterification reaction is promoted by the presence of Zn^2+^ ions at elevated temperatures above the vitrimeric temperature (T_v_), leading to a topological rearrangement.

The effect of the Zn(Ac)_2_ catalyst on the thermal behavior of epoxy vitrimers was assessed. The DSC thermograms of the uncured samples indicate that the catalyst reduces the glass transition temperature of the vitrimeric resins compared to the standard epoxy system. Also, the reaction enthalpy increases with increasing zinc acetate content.

The glass transition temperatures obtained from DMA analyses are higher than those obtained from DSC. The difference is mainly ascribed to the delay in response during the temperature scan in the case of the DMA test due to the larger sample size [48].

The storage modulus at room temperature is also affected by the Zn(Ac)_2_ catalyst, both in the case of system A and B. System B is more rigid than system A, and also exhibits higher damping capacity. By adding Zn(Ac)_2_, this correlation is maintained. At high temperatures above the glass transition temperature, a residual viscous flow is shown. The elastic modulus of the vitrimeric formulations decreases at high temperatures (see inset in Figure 6). The tanδ of vitrimeric systems at 170 °C is significantly higher than corresponding conventional formulations. It increases with the increasing temperature, resembling the typical behavior of thermoplastic polymers [49].

To assess the vitrimer-like nature of epoxy and investigate the flow at high temperatures, creep tests were performed and analyzed by a theoretical model. There are several theoretical models that can be used to analyze the experimental creep curves.

**Figure 12:**
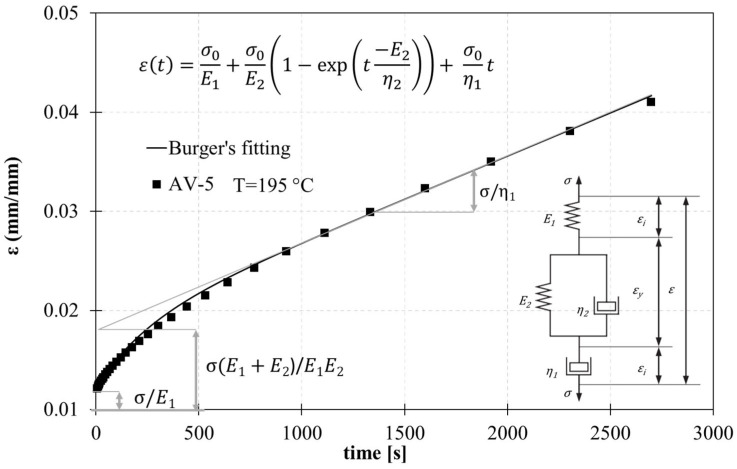
Best fitting of creep data for the AV-5 system at 195 °C by Burger’s viscoelastic model.

In this study, the Burger model was used, which is the association of the Maxwell and Kelvin–Voigt elements [50]. According to Burger’s model, the total strain in the creep is the result of instantaneous deformation and deformation at primary and secondary stages (Figure 12).

The amount of total strain is given by the equation:

(1) $$\varepsilon \left(t\right)=\varepsilon_{i}+\varepsilon_{y}$$

where $\varepsilon \left(t\right)$ is the total strain obtained during the creep test at a particular time *t*, $\varepsilon_{i}$ is the instantaneous deformation, and $\varepsilon_{y}$ is the delay elastic deformation of the Kelvin–Voigt element. Equation (2) can be rewritten as:

(2) $$\varepsilon \left(t\right)=\frac{\sigma_{0}}{E_{1}}+\frac{\sigma_{0}}{E_{2}}\left(1-exp(t\frac{{-E}_{2}}{\eta_{2}}\right)+\frac{\sigma_{0}}{\eta_{1}}t$$

where *σ*_0_ is the applied stress, and *E*_1_ is the modulus of longitudinal elasticity at the initial deformation, which can be recovered once the stress has been removed (Maxwell spring); the constant *η*_1,_ the coefficient of dynamic viscosity, can be identified as the constant rate of stationary creep; *E*_2_ is the stiffness of the amorphous chain/retardant elasticity and is represented by the spring in the Kelvin–Voigt unit, *η*_2_ is the viscosity of the Kelvin–Voigt unit, and the ratio between *η*_2_/*E*_2_ is the retardation time (*τ*). The parameters (*E*_1_, *E*_2_, *η*_1_, and *η*_2_) were evaluated by curve fitting the experimental creep curves with Burger’s fitting. 

**Figure 13:**
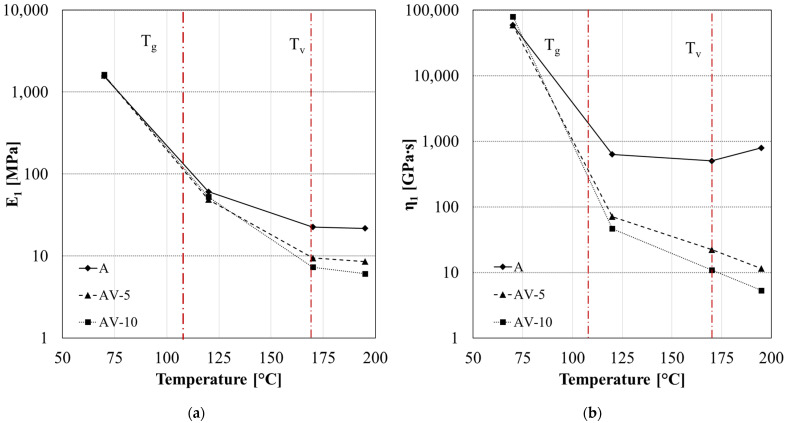
Fitting parameters of creep analysis for system A, AV-5 and AV-10: (**a**) elastic modulus *E*_1_ and (**b**) permanent viscous flow *η*_1_.

**Figure 14:**
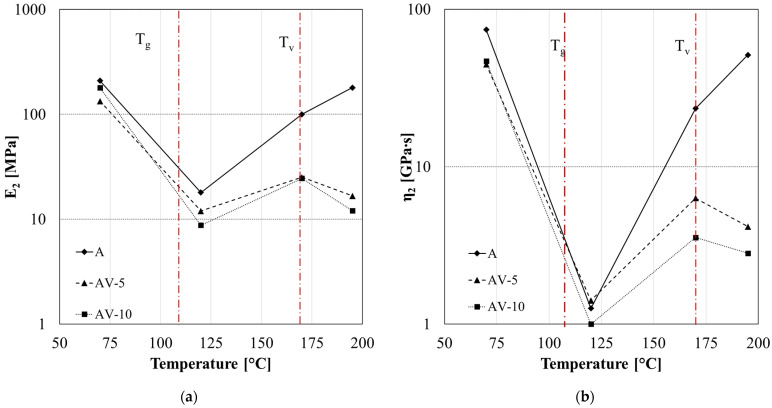
Fitting parameters of creep analysis for system A, AV-5 and AV-10: retardancy elasticity *E*_2_ (**a**) and viscosity *η*_2_ (**b**).

Figure 13 and Figure 14 report the fitting parameters for the creep curves in the case of A-system and its vitrimeric modification at different temperatures (70 °C, 120 °C,170 °C, and 195 °C); the temperature was chosen according to the different regimes: glassy (below T_g_), viscoelastic solid (above T_g_, below T_v_), and liquid viscoelastic (above T_v_).

The elastic modulus E_1_ (Figure 13a) decreases with increasing temperature due to the molecular rearrangement. Unlike standard epoxy system (A), the vitrimeric systems (AV5- and AV-10), above T_v_, exhibit a further reduction of the elastic modulus, resembling the thermoplastic-like behavior. Starting from 170 °C, the vitrimeric epoxy becomes less stiff and the polymer starts to flow, resulting in a progressive decrease in the elastic modulus. The higher the zinc acetate content, the lower the elastic modulus.

*η*_1_ represents the irrecoverable part of the creep deformation, and it is the measure of the residual strain left in the material. Similarly to E_1_, this parameter decreases with the temperature until the T_g_ due to the greater mobility of the molecular chains (Figure 13b). In the case of A systems, it remains constant with increasing temperature due to the thermosetting nature of the material. In the case of vitrimeric systems AV-5 and AV-10, *η*_1_ decreases with the temperature, whereas the standard epoxy achieves a constant value due to the absence of flow. The ability to flow (low *η*_1_ parameter) is related to the presence of the zinc catalyst and increases at higher temperatures after the T_v_ due to further molecular flow induced by the exchange reaction (transesterification). System A shows a slight dependence on the zinc content; in fact, the estimation of *η*_1_ is similar for the AV-5 and the AV-10 resins.

The parameters *E*_2_ (Figure 14a) and *η*_2_ (Figure 14b) represent the retardancy elasticity and viscosity, respectively, and are associated with the stiffness and viscous flow of amorphous polymer chains. The retardancy elasticity and viscosity showed similar dependency on the temperature. Specifically, three different zones can be identified in Figure 14:

(i) T < T_g_

both parameters decrease due to the greater energy absorbed by the active polymer chains, and the viscous slippage of the molecules becomes easier to achieve;

(ii) T_g_ < T < T_v_

the *E*_2_ and *η*_2_ increase with the temperature due to the more significant orientation of polymer chains along the creep loading direction, which results in an orientational hardening; and

(iii) T > T_v_

a further reduction of retardancy parameters is observed only in the case of vitrimeric systems (AV-5 and AV-10) due to the molecular flow induced by the transesterification reaction. This effect depends on the amount of zinc acetate content [51].

### 5.2. Effect of Stoichiometry

**Figure 15:**
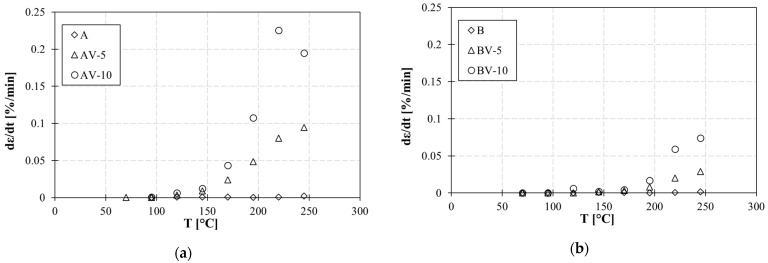
Strain rate at different temperatures for formulation (**a**) A and (**b**) B.

The experimental results showed that the stoichiometry ratio significantly affects the vitrimeric behavior of the modified epoxy. The strain rate (dε/dt), evaluated as the slope of the strain versus time in the last 5 min of the creep test, is higher in the case of AV systems (Figure 15). In a perfectly balanced system (epoxy/acyl ratio equal to 1), the effect of viscoelastic flow is more evident (Figure 15a), compared to the unbalanced system (epoxy/acyl ratio equal to 0.6) (Figure 15b). In other terms, in system A, a lower content of the catalyst is necessary to allow molecular flow with respect to system B, where the high crosslink density of the material limits the molecular mobility of the polymer.

In addition, the strain rate increases with the increasing percentage of zinc acetate and with the temperature due to the mobility of polymer chains promoted by the transesterification reactions.

The higher crosslink density also affects the glass transition temperature, which decreases, increasing the epoxy/acid ratio [52,53,54]. As discussed in Table 2, the highest reaction enthalpy of system B is linked to the highest crosslink density. On the contrary, the lowest reaction enthalpy of system A leads to the lowest crosslink density.

Also, the tensile strength (Figure 5) of the systems is affected by the epoxy/acid ratio, decreasing with the epoxy/acid ratio.

Even though system B exhibited better mechanical performance in terms of elastic modulus and tensile strength, the polymer molecular mobility at high temperatures is strongly limited, thus inhibiting the self-healing capacity and negatively affecting the recovery and recycling of the material.

**Figure 16:**
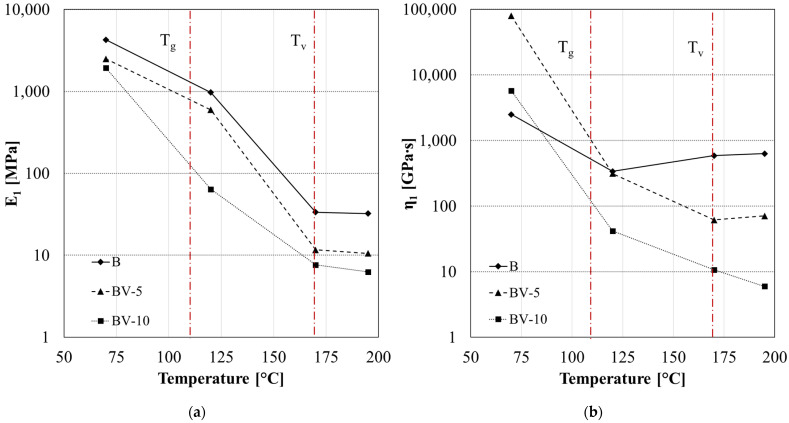
Fitting parameters of creep analysis for system B, BV-5 and BV-10: (**a**) elastic modulus *E*_1_ and (**b**) permanent viscous flow *η*_1_.

Analyzing the creep curve also in the case of system B (unbalanced), standard and vitrimeric, it is noticed that the elastic modulus decreases with increasing temperature (Figure 16a). Also, in this case, the BV-5 and BV-10 systems showed a further decrease in the modulus after the T_v_ due to the polymer flowing.

Observing Figure 16b, it appears that there is a lower ability to flow compared to the vitrimeric system AV-5 and AV-10. In fact, the decrease after T_v_ in parameter *η*_1_ is found only for the sample with 10% zinc acetate. This reflects the effect of the stochiometric ratio on the polymeric chain mobility.

**Figure 17:**
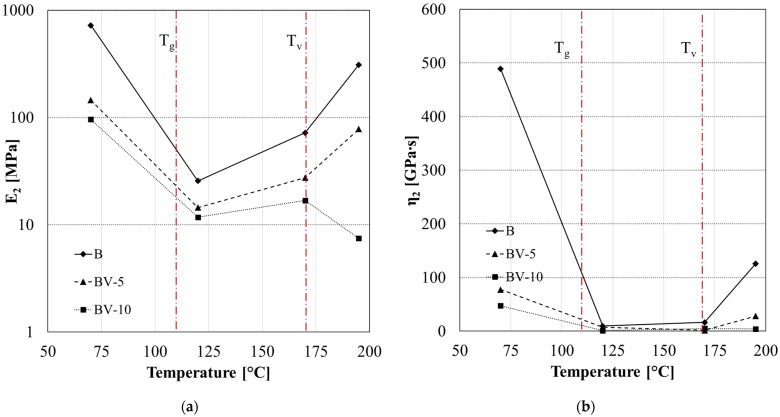
Fitting parameters of creep analysis for system B, BV-5 and BV-10: retardancy elasticity *E*_2_ (**a**) and viscosity *η*_2_ (**b**).

The retardancy parameters trend confirmed the latter behavior. In fact, in the third zone (T > T_v_), a decrease in *E*_2_ (Figure 17a) and *η*_2_ (Figure 17b) occurs only in the case of BV-10.

## 6. Conclusions

In this work, a commercially available resin was considered for modification by a metal catalyst able to induce a covalent adaptable network, which would promote the reuse and recycling of the epoxy system and its composites.

The effect of the epoxy-to-acyl group ratio (0.6 and 1) and zinc acetate catalyst content (0, 5 and 10%) were investigated and discussed. Thermomechanical and creep tests demonstrated that the catalyst induces the vitrimeric behavior:

- The glass transition temperature of the vitrimeric system slightly reduces by 5% and 15% for polymers AV5 and AV10, respectively, compared to A, and increases by 4% and 13% for polymers BV5 and BV10, respectively, compared to B.
- The storage modulus and damping ratio trends with temperature of the vitrimeric systems indicate that at low temperatures, the polymer behaves like a thermoset, whereas at high temperatures, above T_g_, the polymer flows resemble the behavior of a thermoplastic polymer.
- Evident creep of samples subjected to tensile load was found at different temperatures. The presence of metallic ions strongly catalyzes the ester-interchange reaction, particularly in the balanced systems (AV5 and AV10).
- The creep analysis according to Burger’s model showed that the elastic and viscous parameters are affected by the epoxy/acyl group and the catalyst content. The highest flow at a high temperature was obtained for the balanced stoichiometric ratio and 10% of zinc acetate due to the higher molecular mobility promoted by transesterification reactions.
- The induced flow at temperatures above T_v_ enables the recyclability of the polymer and its composites. By applying adequate temperature and pressure to the material, it can be reshaped and reformed, maintaining a similar performance to the pristine material.

## Figures and Tables

**Table 1:** Cross-linked sample composition.

Sample Codex	LY 3508 [phr]	Aradur 917-1 [phr]	960-1 acc. [phr]	Epoxy/Acyl Ratio, r [-]	ZnAc_2_ * [%]
A	100	40	3	1.0	0
AV-5	100	40	3	1.0	5
AV-10	100	40	3	1.0	10
B	100	70	3	0.6	0
BV-5	100	70	3	0.6	5
BV-10	100	70	3	0.6	10

* ZnAc_2_ percentage is calculated to the total acyl groups.

**Table 2:** Results of TGA and DSC analyses.

Description	T @ 10% Decomposition [°C]	T @ Maximum Decomposition [°C]	Char Yield @800 °C [wt%]	T_g, DSC_ [°C]	Reaction Enthalpy [J/g]	T_peak_ [°C]
A	369	410	7.04	111.2	216.6 ± 1.2	130 ± 5
AV-5	333	405	13.13	105.1	242.5 ± 1.6	140 ± 1
AV-10	325	402	15.15	94.3	270.3 ± 2.0	139 ± 2
B	371	416	5.44	111.5	282.7 ± 1.1	137 ± 4
BV-5	337	413	12.55	115.6	287.5 ± 1.9	150 ± 5
BV-10	341	414	13.98	126.3	280.2 ± 2.6	145 ± 3

**Table 3:** Ultimate tensile stress and strain and elastic modulus for A and B systems.

	Elastic Modulus [MPa]	Ultimate Strain [mm/mm]	Ultimate Stress [MPa]
A	2704 ± 5	0.028 ± 0.002	68.3 ± 0.7
AV-5	2596 ± 8	0.027 ± 0.003	61.5 ± 0.6
AV-10	2087 ± 7	0.024 ± 0.005	57.2 ± 0.5
B	3059 ± 11	0.036 ± 0.004	83.2 ± 1.0
BV-5	2998 ± 6	0.027 ± 0.004	74.2 ± 0.7
BV-10	3018 ± 5	0.020 ± 0.003	58.5 ± 0.6

**Table 4:** Results of DMA analysis at 35 °C and 170 °C.

		@35 °C			@170 °C		
	T_g, DMA_ [°C]	E″ [Mpa]	E″ [Mpa]	Tanδ [-]	E′ [Mpa]	E″ [Mpa]	Tanδ [-]
A	105.6 ± 1.1	1899 ± 33	22.1 ± 0.3	0.0116 ± 0.0002	5.14	0.02	0.004
AV-5	108.8 ± 0.9	2221 ± 56	28.5 ± 0.5	0.0128 ± 0.0001	12.08	1.43	0.118
AV-10	101.0 ± 0.7	2484 ± 47	33.4 ± 0.3	0.0135 ± 0.0003	11.89	3.08	0.259
B	117.3 ± 0.8	1867 ± 29	26.7 ± 0.2	0.0143 ± 0.0003	9.20	0.02	0.002
BV-5	119.1 ± 1.5	2369 ± 21	31.5 ± 0.4	0.0133 ± 0.0004	10.88	1.61	0.148
BV-10	113.6 ± 0.6	2698 ± 37	30.7 ± 0.1	0.0114 ± 0.0002	6.70	3.48	0.518

## Data Availability

The data presented in this study are available in the article.
